# Phosphorylation of Shiftless by casein kinase 1 δ/ε is required for its antiviral activity

**DOI:** 10.1128/jvi.01864-25

**Published:** 2025-12-02

**Authors:** Yongle Wang, Shaozu Fu, Xinlu Wang, Guangxia Gao

**Affiliations:** 1 State Key Laboratory of Biomacromolecules, Institute of Biophysics, Chinese Academy of Sciences 318333 , Beijing, China; 2 University of Chinese Academy of Sciences, Beijing, China; The Ohio State University, Columbus, Ohio, USA

**Keywords:** antiviral factor, -1 programmed ribosomal frameshifting, casein kinase 1, phosphorylation, Shiftless

## Abstract

Shiftless (SHFL) is a host antiviral factor that inhibits the viral −1 programmed ribosomal frameshifting (−1PRF). How the antiviral activity of SHFL is regulated is not known. Here, we report that the phosphorylation of SHFL by the casein kinase 1 delta (CK1δ) and epsilon (CK1ε) regulates its antiviral activity. The casein kinases were identified as SHFL-interacting proteins. Downregulation of the expression of the endogenous casein kinases or pharmacological inhibition of kinase activity significantly impaired the activity of SHFL to inhibit HIV-1 -1PRF and viral production. The residues T250 and T253 were identified as critical phosphorylation sites; substitution of either of the residues with alanine, resulting in the mutants SHFL-T250A or SHFL-T253A, abolished the antiviral activity of SHFL against HIV-1. The T250A mutation did not affect SHFL interaction with target RNA or the ribosomal proteins uL5 and eS31 but significantly reduced its interaction with IGF2BP proteins. Furthermore, SHFL-T250A displayed reduced association with the actively translating polysomes. These results are consistent with the notion that SHFL interaction with ribosomes is critical for its activity to inhibit −1PRF. Taken together, our results indicate that phosphorylation of SHFL by CK1δ/ε is required for its antiviral activity.

**IMPORTANCE:**

Innate immune responses and antiviral defense mechanisms are regulated through a variety of mechanisms, among which post-translational modifications, especially phosphorylation, play important roles. Deciphering the regulatory mechanisms helps understand the innate immune responses more comprehensively. SHFL is an interferon-stimulated host antiviral factor that inhibits the replication of multiple viruses. The present study revealed that phosphorylation of SHFL by casein kinase 1 δ/ε is required for its antiviral activity against HIV-1. These results provide an additional example for how immune responses are regulated. Furthermore, given that SHFL inhibits −1PRF, the present studies provide tools for further exploring the mechanisms of −1PRF.

## INTRODUCTION

Shiftless (SHFL) is an interferon-stimulated host antiviral factor that inhibits the replication of multiple viruses. It inhibits the −1 ribosomal frameshifting (−1PRF) of HIV-1 and thus inhibits the replication of the virus (1). In addition to HIV-1, SHFL has been reported to inhibit the −1PRF of other viruses including SARS-CoV-2 and Japanese encephalitis virus (2–4). SHFL has also been reported to inhibit the replication of some viruses that do not have obvious −1PRF (5), but the underlying mechanism is not yet elucidated. Recent reports suggest that SHFL also participates in ribosome surveillance (6).

−1PRF is a translational recoding mechanism widely used by viruses to expand their genome coding capacity and regulate viral protein expression. The frameshifting occurs at specific −1PRF site, which typically consist of slippery sequences and downstream RNA high-order structures such as pseudoknots or stem-loops. The translating ribosomes pause at the −1PRF site because of the downstream high-order structure block. Although most ribosomes manage to pass the block to continue translation in the original frame, some ribosomes shift one nucleotide backward at the slippery sequence and continue translation in the −1 frame (7). Precise control of −1PRF efficiency is essential for viral replication (8, 9). For instance, HIV-1 uses −1PRF to synthesize Gag (0 frame) and Gag-Pol (−1 frame) with the frameshifting efficiency 5%–10%, resulting in Gag to Gag-Pol ratio to be 10-20:1. Such a controlled ratio is critical for HIV-1 virion assembly and replication (10–12). SHFL interacts directly with the −1PRF signals of target mRNAs and with ribosomal proteins uL5 and eS31 (1, 13). It was proposed that SHFL interaction with the translating ribosomes and −1PRF signals causes ribosome stalling, thus inhibiting frameshifting (1).

Phosphorylation is a post-translational modification mechanism that regulates a variety of biological processes by modulating protein activity, localization, and protein-protein interactions (14, 15). This process is controlled by approximately 568 kinases and 156 phosphatases in human cells (16). Among these kinases, casein kinase 1 (CK1) is a conserved serine/threonine kinase family comprising seven isoforms (α, β, γ1-3, δ, and ε) (17, 18). CK1δ and CK1ε, two closely related isoforms with overlapping biological functions, regulate multiple biological processes such as circadian rhythms (19, 20), Wnt signaling (21), protein translation (22), and 40S ribosome biogenesis (23). CK1 preferentially phosphorylates substrates that contain a priming phosphate and typically recognizes a consensus sequence of pS/pT-X-X-S*/T*, where X represents any amino acid and S*/T* denotes the target phosphorylation site. Certain substrates can be directly phosphorylated by CK1 without priming (24–28). It has been reported that CK1-interacting proteins are prone to be phosphorylated, and such interactions can serve as supporting evidence for the identification of CK1 substrates (27).

Here, we identified CK1δ and CK1ε as SHFL-interacting proteins. SHFL phosphorylation by CK1δ/ε is required for its ability to inhibit −1PRF and HIV-1 replication. We identified the T250 and T253 of SHFL as critical phosphorylation sites by CK1δ/ε. The phosphorylation at T250 and T253 is required for its antiviral activity against HIV-1.

## RESULTS

### SHFL interacts with CK1δ/ε

**Fig 1:**
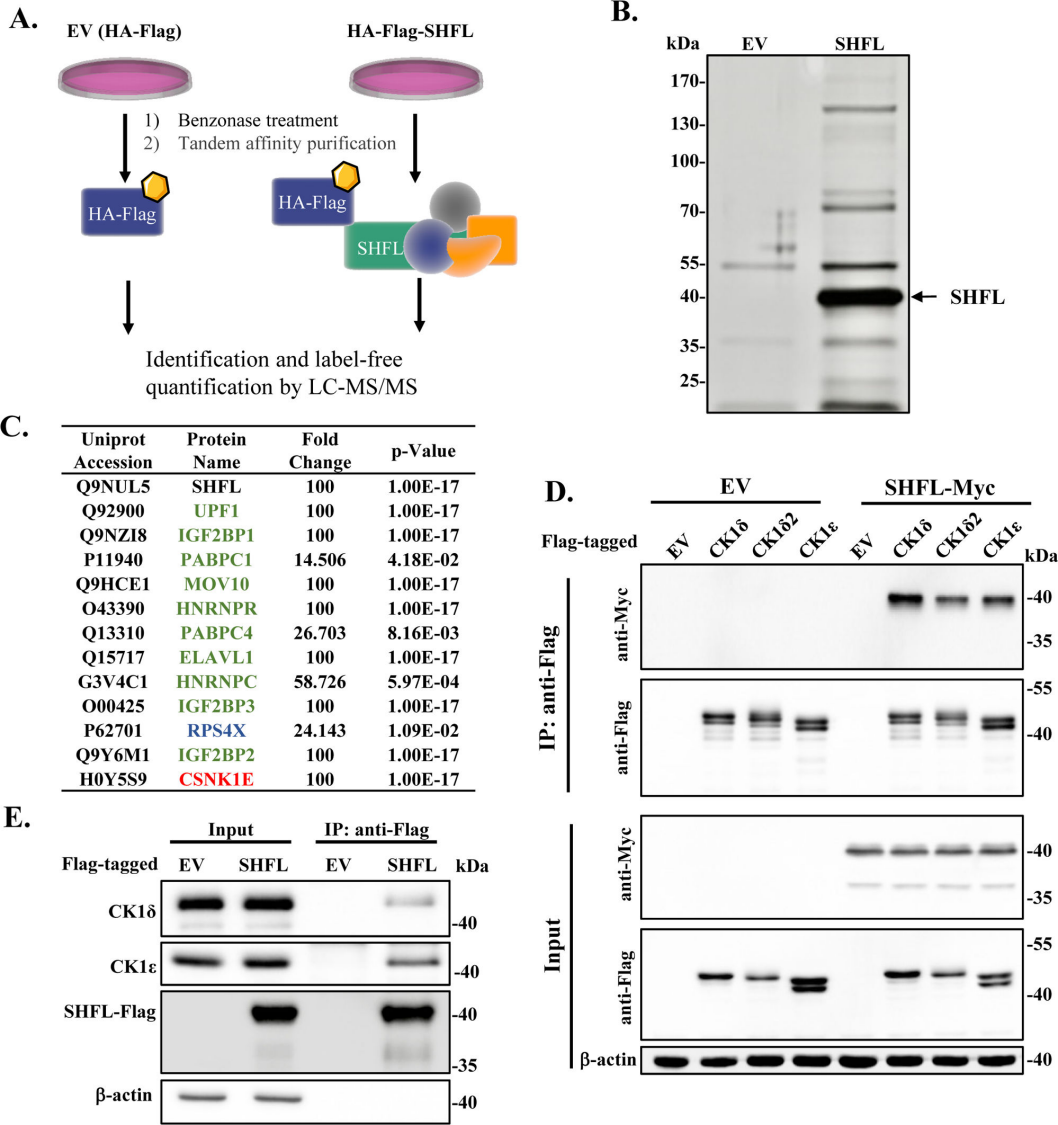
Identification of casein kinase 1 as a SHFL-interacting protein. (**A**) Schematic illustration of the experimental procedure. (**B**) Silver staining detection of TAP-purified SHFL-associated proteins. HEK293 cells that express HA-Flag-SHFL in a doxycycline-inducible manner were treated with doxycycline at 1 µg/mL for 24 h to induce SHFL expression. The cell lysates were subjected to HA-Flag tandem affinity purification. Data shown are representative of three independent experiments. (**C**) List of representative SHFL-interacting proteins identified by the TAP-MS analysis. Fold change was calculated using Proteome Discoverer (version 2.4.1.15) with the Pairwise Ratio Based method. The maximum allowed fold change was set to 100. Significance (*P*-value) was calculated from three independent experiments. (**D**) 293T cells were transiently transfected with constructs expressing the Flag-tagged CK1 isoform indicated and Myc-tagged SHFL. At 48 h post-transfection, cell lysates were immunoprecipitated with anti-Flag affinity gel and subjected to western analysis. Data presented are representative of three independent experiments. (**E**) Flag-tagged SHFL was transiently expressed in 293T cells, immunoprecipitated with anti-Flag affinity gel, and subjected to western analysis. The endogenous CK1δ and CK1ε were detected with specific antibodies. Data presented are representative of two independent experiments.

In an attempt to better understand the mechanisms underlying the antiviral activity of SHFL, we set out to use HA-Flag tandem affinity purification coupled with mass spectrometry (TAP-MS) to identify SHFL-interacting proteins (Fig. 1A). HA-Flag-SHFL was expressed in HEK293 cells in a doxycycline-inducible manner. The cell lysates were first treated with the nuclease Benzonase to disrupt RNA- or DNA-mediated protein-protein interactions. The proteins were sequentially purified with anti-HA and anti-Flag conjugated beads. A fraction of the purified proteins was subjected to mass spectrometry analysis and the rest was analyzed by SDS-PAGE. Silver staining analysis revealed multiple distinct protein bands specific to the SHFL sample (Fig. 1B). Mass spectrometry analysis combined with label-free quantification identified multiple SHFL-associated proteins, including RNA-binding proteins, a ribosomal protein, and CK1ε (Fig. 1C). We focused on CK1 in the following studies.

Although CK1δ was not detected in the TAP-MS analysis, we speculated that SHFL may interact with both CK1δ and CK1ε, considering that they share substantial sequence similarity and functional redundancy and that the rigorous TAP procedure might disrupt weak or transient interactions (27, 29). Two CK1δ isoforms have been identified, CK1δ and CK1δ2, with CK1δ2 lacking six amino acids at the C-terminus (30). The interactions of CK1ε, CK1δ, and CK1δ2 with SHFL were analyzed by the co-immunoprecipitation (Co-IP) assay. Flag-tagged CK1 isoforms and Myc-tagged SHFL were co-expressed in 293T cells, followed by immunoprecipitation using anti-Flag affinity gel. The nuclease Benzonase was added into the cell lysates to disrupt RNA- or DNA-mediated protein interactions. SHFL co-precipitated with all the CK1 isoforms (Fig. 1D).

To assess the interaction between SHFL and endogenous CK1δ/ε, Flag-tagged SHFL was transiently expressed in 293T cells. Cell lysates were immunoprecipitated with anti-Flag affinity gel and analyzed by western blotting. Both CK1δ and CK1ε co-precipitated with SHFL (Fig. 1E), further demonstrating the interaction between SHFL and CK1δ/ε.

### CK1δ/ε phosphorylates SHFL

**Fig 2:**
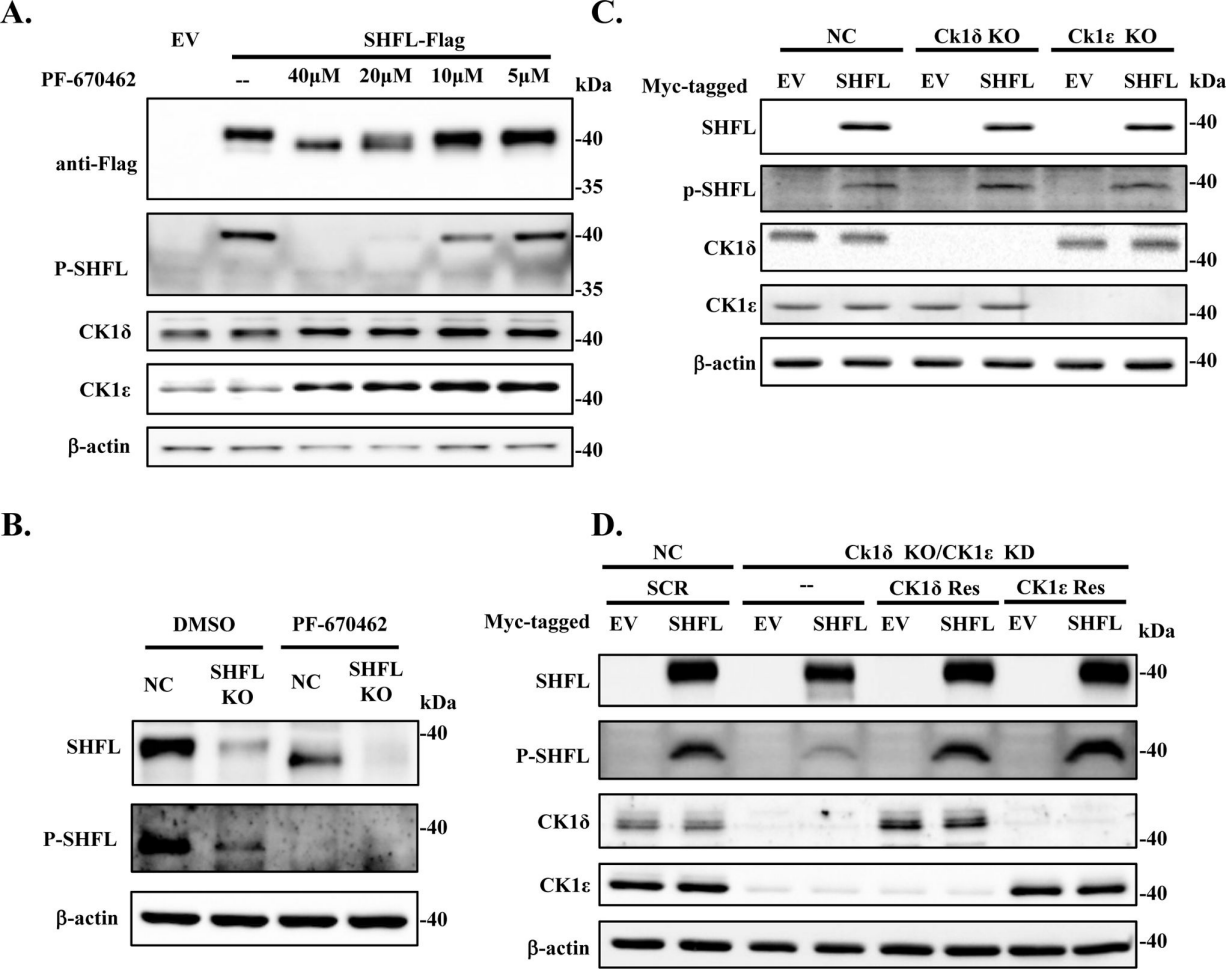
Downregulation or inhibition of the kinase activity of CK1δ/ε reduces SHFL phosphorylation. (**A**) HEK293 cells transiently expressing SHFL-Flag were cultured in medium containing increasing concentrations of CK1 inhibitor PF-670462 for 24 h and then subjected to western analysis. SHFL was detected using anti-Flag antibody or the antisera that specifically recognize SHFL phosphorylated at T250 and T253 (P-SHFL). The endogenous CK1δ and CK1ε were detected with specific antibodies. Data presented are representative of three independent experiments. (**B**) SHFL knockout (KO) and control (NC) THP-1 cells were treated with 40 µM PF-670462. The endogenous SHFL was immunoprecipitated with SHFL-specific polyclonal antibody and subjected to western analysis using the polyclonal antibody or the antisera that specifically recognize SHFL phosphorylated at T250 and T253 (P-SHFL). β-actin in the input samples served as a loading control. Data are representative of two independent experiments. (**C**) The HEK293 cells in which CK1δ or CK1ε was knocked out were transfected with a plasmid expressing Myc-tagged SHFL. At 24 h post-transfection, cell lysates were subjected to western analysis. Data presented are representative of three independent experiments. NC, control HEK293 cells. (**D**) CK1δ knockout HEK293 cells were transfected with siRNA targeting CK1ε (CK1ε KD), with or without co-transfection of a CK1ε or CK1δ rescue construct (CK1ε Res or CK1δ Res). As a control, HEK293 NC cells were transfected with scrambled siRNA (SCR). The cells were then transfected with a plasmid expressing Myc-tagged SHFL. At 24 h post-transfection, cell lysates were analyzed by western blotting as described above. Data shown are representative of three independent experiments.

The interaction between a kinase and its binding partner often suggests a potential kinase-substrate relationship (31). We reasoned that SHFL may be a phosphorylation substrate of CK1δ and CK1ε. To examine whether CK1δ/ε is involved in SHFL phosphorylation, HEK293 cells transiently expressing Flag-tagged SHFL were treated with increasing concentrations of CK1δ/ε-specific inhibitor PF-670462 (32). The phosphorylation of SHFL was monitored by its electrophoretic mobility shift on SDS-PAGE. The protein with less phosphorylation is expected to move faster. Indeed, SHFL expressed in the cells treated with the inhibitor moved faster than that expressed in the mock-treated cells (Fig. 2A). To show that the faster-moving SHFL was less phosphorylated, we generated antisera for western analysis that specifically recognize SHFL phosphorylated at T250 and T253, which were later identified as critical phosphorylation sites for CK1δ/ε (see below for more information). Consistent with the above mobility shift results, treatment with the inhibitor reduced the phosphorylation of SHFL in a dose-dependent manner (Fig. 2A).

We next examined whether the endogenous SHFL is phosphorylated. The SHFL knockout (KO) or control (NC) THP-1 cells were treated with interferon-alpha to upregulate the expression of the endogenous SHFL (1, 33). The cells were treated with DMSO or PF-670462. Since the phospho-SHFL-specific antisera were not sensitive enough to detect the endogenous SHFL in the cell lysate, SHFL was first immunoprecipitated with SHFL-specific polyclonal antibody and then analyzed by western blotting. Treatment with PF-670462 led to increased electrophoretic mobility of SHFL (Fig. 2B). Consistently, phosphorylation of the endogenous SHFL was barely detected in the PF-670462-treated cells (Fig. 2B). These results further show that SHFL is phosphorylated, and the phosphorylation can be suppressed by the CK1δ/ε inhibitor PF-670462.

To test which kinase is involved in the phosphorylation of SHFL, we generated CK1δ and CK1ε knockout HEK293 cells. Myc-tagged SHFL was transiently expressed in these cells and analyzed by western blotting. Knockout of either kinase alone did not alter the electrophoretic mobility of SHFL or its phosphorylation at T250 and T253 (Fig. 2C), suggesting that CK1δ and CK1ε function redundantly. We thus downregulated the expression of both CK1δ and CK1ε in HEK293 cells. Since simultaneous knockout of these two kinases was lethal to the cells, CK1δ was knocked out by the CRISPR/Cas9 method, and CK1ε was knocked down by the siRNA method. Myc-tagged SHFL was transiently expressed in these cells and analyzed by western blotting. Consistent with the inhibitor treatment results, downregulation of CK1δ/ε rendered SHFL moving faster, indicative of reduced phosphorylation. Furthermore, the phospho-specific antisera detected less SHFL phosphorylated at T250 and T253 in the cells in which CK1δ and CK1ε were downregulated. To confirm the specificity of the downregulation, a CK1δ or CK1ε-expressing rescue construct was used, which was not targeted by the sgRNA or siRNA. Expression of the rescue construct restored SHFL migration pattern and phosphorylation level (Fig. 2D). Collectively, these results indicate that CK1δ and CK1ε are required for the phosphorylation of SHFL.

### CK1δ/ε phosphorylation of SHFL is required for its antiviral activity

**Fig 3:**
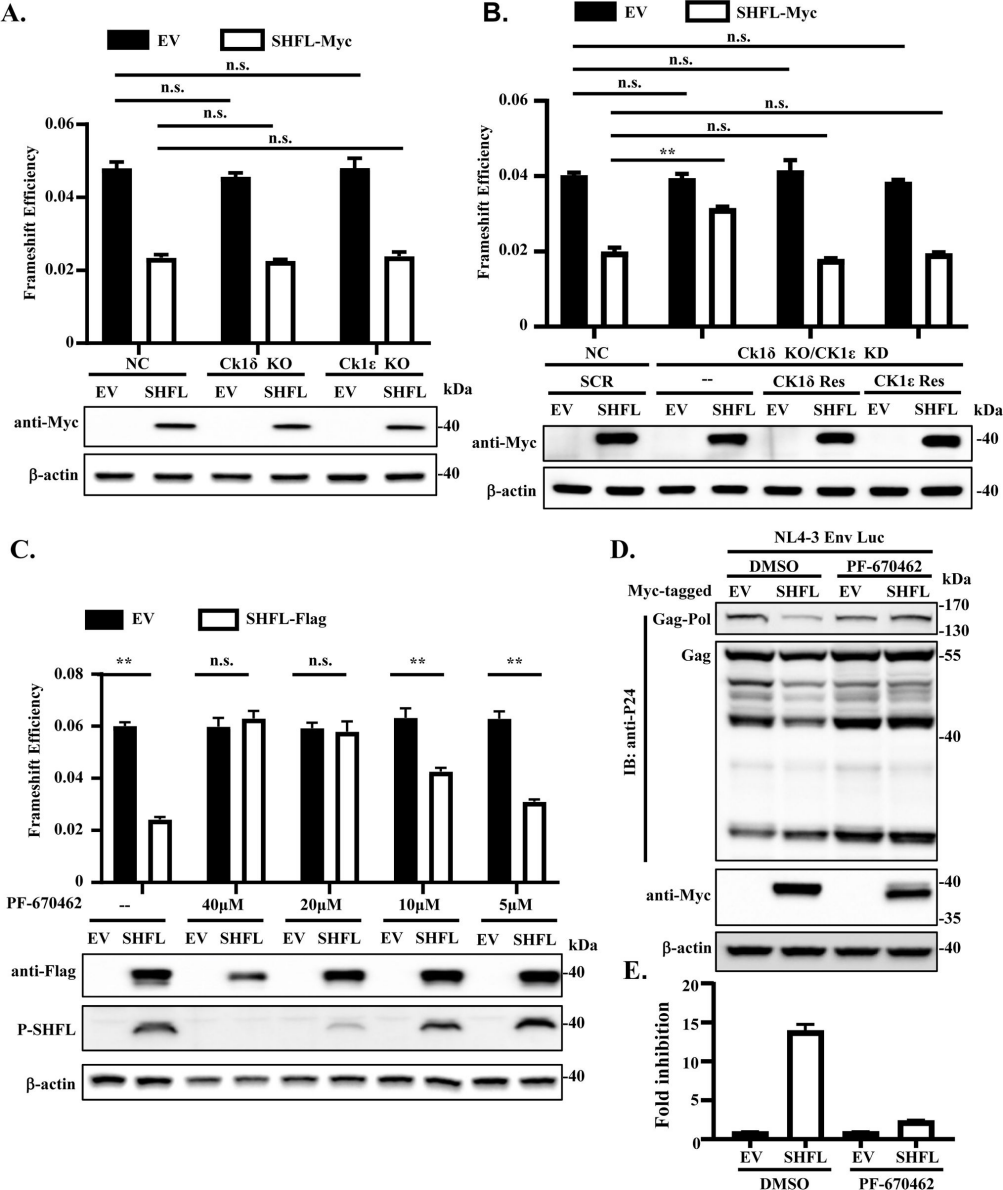
Downregulation or inhibition of the kinase activity of CK1δ/ε impairs the antiviral activity of SHFL. (**A**) HEK293 cells in which CK1δ or CK1ε was knocked out were transfected with the −1PRF dual-luciferase reporters and a construct expressing Myc-tagged SHFL. HEK293 NC cells were used as a control. At 24 h post-transfection, the cells were lysed, and luciferase activities were measured. The frameshift efficiency was calculated as the ratio of Fluc/Rluc expressed from the HIV (−1) reporter divided by that from the HIV (0) reporter. Data presented are means ± SD of two independent experiments. n.s. denotes not significant (*P* > 0.05). (**B**) CK1δ knockout HEK293 cells were transfected with the siRNA targeting CK1ε, with or without co-transfection of a CK1ε or CK1δ rescue construct (CK1ε Res or CK1δ Res). As a control, HEK293 NC cells were transfected with scrambled siRNA. The cells were transfected with the −1PRF dual-luciferase reporter and a construct expressing Myc-tagged SHFL. At 24 h post-transfection, the cells were lysed, and the luciferase activities were measured. The frameshift efficiency was calculated as in (**A**). Data presented are means ± SD of two independent experiments. ** denotes *P* < 0.01; n.s. denotes not significant (*P* > 0.05). (**C**) 293T cells were co-transfected with the dual-luciferase reporter with an empty vector (EV) or a construct expressing Flag-tagged SHFL. The cells were cultured in medium containing increasing concentrations of PF-670462 for 24 h. The frameshift efficiency, SHFL expression, and phosphorylation status were analyzed as described above. Data presented are means ± SD of two independent experiments. ** denotes *P* < 0.01; n.s. denotes not significant. (**D and E**) 293T cells were transfected with the HIV-1 producing vector pNL4-3 Env-Luc, together with an EV or a construct expressing Myc-tagged SHFL. The cells were cultured in medium containing either DMSO or PF-670462. At 48 h post-transfection, the culture supernatants were collected and used to infect HOS-CD4/CCR5 recipient cells. Producer cells were lysed and luciferase activity was measured. HIV-1 Gag and Gag-Pol expression in the producer cells was analyzed by western blotting (**D**). At 48 h postinfection, recipient cells were lysed and luciferase activity was measured. The luciferase activity in the recipient cells was normalized with the luciferase activity in the producer cells. Fold inhibition was calculated as the normalized luciferase activity from the control cells divided by that from the SHFL-expressing cells (**E**). Data presented are means ± SD of two independent experiments. n.s. denotes not significant.

We next analyzed the function of CK1δ/ε in SHFL inhibition of −1PRF. A well-established HIV-1 dual-luciferase reporter system was employed to assess −1PRF efficiency (1). In this system, the −1PRF signal from HIV-1 was inserted between the coding sequences of Renilla luciferase (Rluc) and Firefly luciferase (Fluc) in the pDual-HIV(−1) reporter, with Fluc in the −1 reading frame. The reporter pDual-HIV(0) was used as a control, in which a nucleotide was added in the slippery sequence such that Rluc and Fluc are in the same reading frame. The −1PRF efficiency was calculated as the Fluc/Rluc ratio from pDual-HIV(−1) divided by that from pDual-HIV(0). To assess the role of CK1δ/ε in SHFL inhibition of −1PRF, we first examined whether the knockout of CK1δ or CK1ε affected SHFL function. HEK293 cells were co-transfected with the HIV-1 dual-luciferase −1PRF reporters and a construct expressing Myc-tagged SHFL, and frameshifting efficiency was measured in the presence or absence of SHFL. Knockout of either kinase alone did not obviously affect SHFL suppression of −1PRF (Fig. 3A), consistent with the above observation that phosphorylation at T250 and T253 was not altered by the knockout of either CK1δ or CK1ε alone. We next analyzed SHFL activity in the cells in which CK1δ was knocked out and CK1ε was knocked down. In the control cells, SHFL markedly suppressed −1PRF efficiency (Fig. 3B). CK1δ/ε downregulation significantly impaired the inhibitory effect, and expression of the CK1δ or CK1ε rescue construct restored the ability of SHFL to suppress −1PRF (Fig. 3B). In line with these results, the CK1δ/ε inhibitor PF-670462 attenuated SHFL inhibition of frameshifting in a dose-dependent manner, and the SHFL activity well correlated with its phosphorylation level (Fig. 3C).

We further analyzed the function of CK1δ/ε in regulating the antiviral activity of SHFL against HIV-1. The pNL4-3 Env-Luc plasmid is a modified HIV-1 NL4-3 construct in which the coding sequence of Firefly luciferase is inserted in-frame within the coding sequence of Nef (33). Myc-tagged SHFL was co-expressed with pNL4-3 Env-Luc in 293T cells and treated with either DMSO or the CK1δ/ε inhibitor PF-670462. The expression levels of the viral proteins in these cells were measured by western analysis. The culture supernatants were used to infect recipient cells to evaluate the production of the virus. In the DMSO-treated cells, SHFL markedly reduced Gag-Pol protein level (Fig. 3D). The inhibitory effect was nearly abolished upon PF-670462 treatment (Fig. 3D). Without PF-670462 treatment, SHFL significantly inhibited the production of the virus (Fig. 3E). In comparison, PF-670462 treatment dramatically compromised the antiviral activity of SHFL (Fig. 3E). These results further demonstrate that CK1δ/ε-mediated phosphorylation is required for the antiviral activity of SHFL against HIV-1.

### CK1δ/ε-mediated phosphorylation at T250 and T253 is essential for the antiviral activity of SHFL

**Fig 4:**
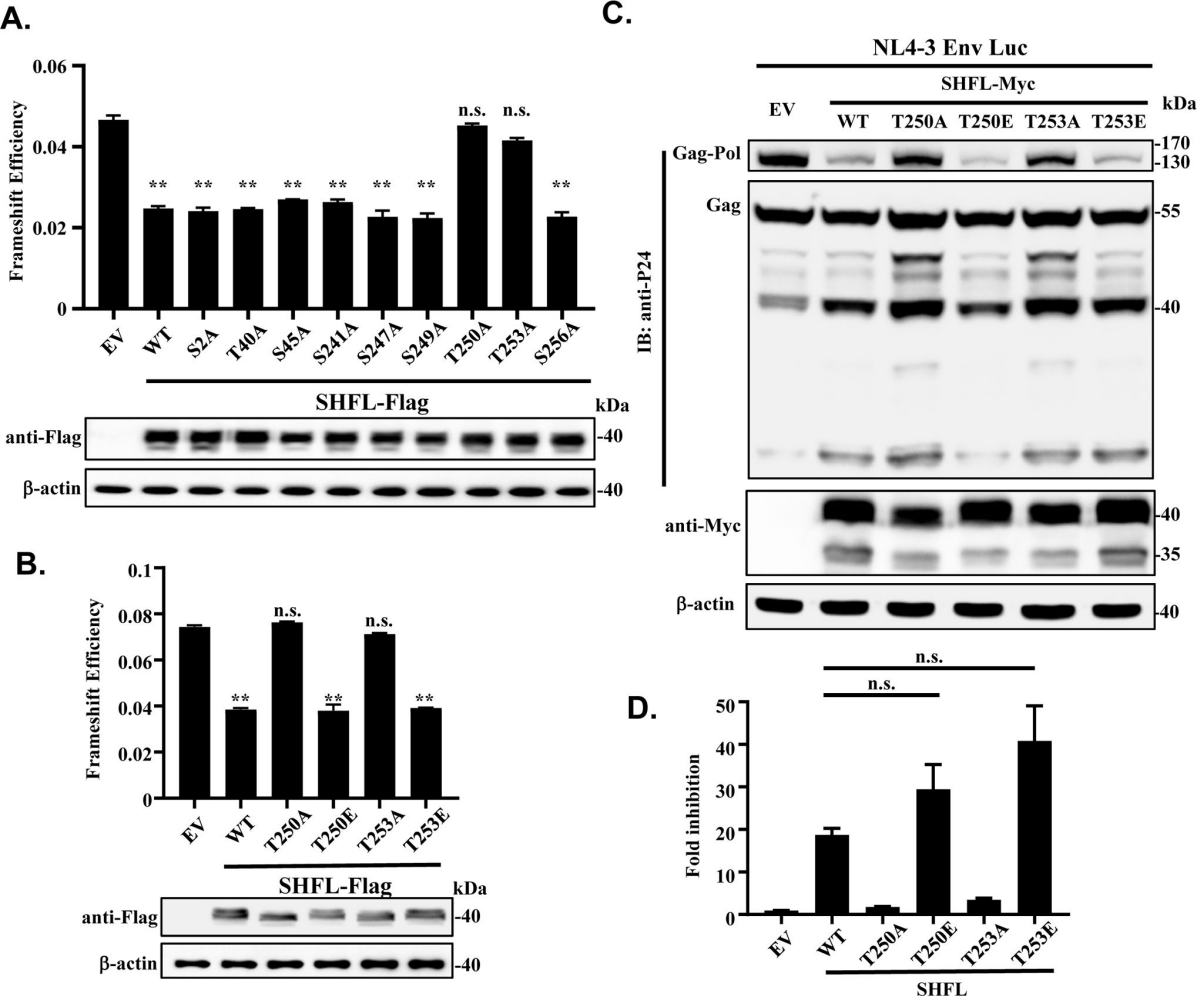
Phosphorylation at T250 and T253 is required for SHFL inhibition of frameshifting and HIV-1 production. (**A and B**) 293T cells were co-transfected with the dual-luciferase reporter and a construct expressing the Flag-tagged SHFL mutant indicated. At 24 h post-transfection, luciferase activities were measured and frameshift efficiency was calculated as described above. Data presented are means ± SD of three independent experiments. ** denotes *P* < 0.01; n.s. denotes not significant. EV, empty vector; WT, wild-type SHFL. (**C and D**) 293T cells were co-transfected with the HIV-1 vector pNL4-3 Env-Luc and a plasmid expressing the Myc-tagged SHFL indicated. At 48 h post-transfection, culture supernatants were collected and used to infect HOS-CD4/CCR5 recipient cells. The effects on Gag-Pol expression and fold inhibition of SHFL were analyzed as described in the legend to Fig. 3D and E. Data presented are means ± SD of three independent experiments.

We next investigated which phosphorylation sites in SHFL are functionally important for the regulatory activity. The candidate phosphorylation residues from the PhosphoSitePlus database were each substituted with alanine. The mutants were tested for their ability to inhibit −1PRF using the dual-luciferase reporters in 293T cells. Although most mutations had little effect, the T250A and T253A mutations nearly abolished the function of SHFL to inhibit frameshifting (Fig. 4A). These results and the foregoing results suggested that the phosphorylation at T250 and T253 by CK1δ/ε is essential for SHFL to inhibit −1PRF. To substantiate this notion, we generated two phosphomimetic mutants of SHFL, T250E and T253E, and analyzed their ability to inhibit −1PRF. As expected, these two mutants displayed comparable activity as the wild-type SHFL (Fig. 4B).

To demonstrate that the phosphorylation of SHFL at T250 and T253 is important for its antiviral activity, we analyzed the effect of these mutants on the expression of HIV-1 Gag-pol and virus production. 293T cells were transiently co-transfected with pNL4-3 Env-Luc and Myc-tagged SHFL variants. Consistent with the previous results, the wild-type SHFL inhibited the expression of Gag-pol (Fig. 4C) and the virus production (Fig. 4D). Although the antiviral activity of the SHFL-T250A and -T253A mutants was markedly reduced, the SHFL-T250E and -T253E mutants exhibited comparable antiviral activity as the wild-type protein (Fig. 4C and D). These results indicate that the phosphorylation at T250 and T253 is critical for the antiviral activity of SHFL. Considering that the SHFL-T250A and -T253A mutants displayed very similar phenotypes, we focused on the SHFL-T250A mutant in the following studies.

### Phosphorylation at T250 modulates SHFL association with polysomes

**Fig 5:**
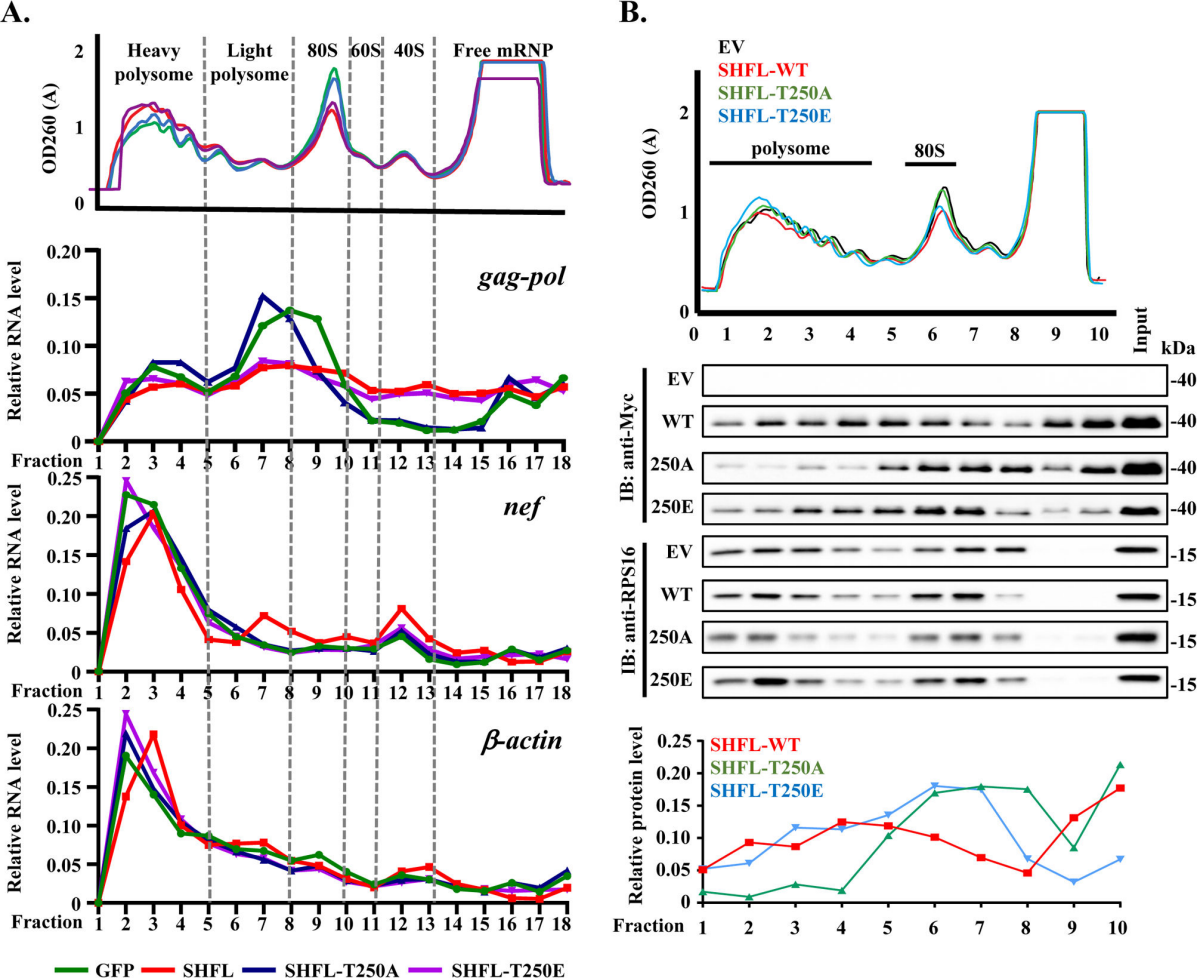
The T250A mutation reduces SHFL association with polysomes. (**A**) HOS-CD4/CCR5 cells expressing the Myc-tagged protein indicated in a doxycycline-inducible manner were infected with VSV-G pseudotyped NL4-3 GFP for 3 h and treated with doxycycline to induce protein expression. At 24 h postinfection, the cell lysates were fractionated through a 10%–50% sucrose gradient with continuous monitoring of absorbance at 260 nm (upper panel). The RNA levels in each fraction were measured by RT-qPCR. Relative RNA level was calculated as the RNA level in each fraction divided by the total RNA level in all the fractions. Data presented are representative of two independent experiments. (**B**) 293T cells were transfected with an empty vector (EV) or a plasmid expressing the Myc-tagged SHFL indicated. At 24 h post-transfection, the cells were treated with cycloheximide and lysed in polysome lysis buffer. The cell lysates were fractionated through a 10%–50% sucrose gradient with continuous monitoring of absorbance at 260 nm (upper panel). The protein levels in each fraction were measured by western analysis (middle panel). Relative protein levels of SHFL variants in the middle panel were quantified using ImageJ by dividing the signal intensity of each fraction by the total signal intensity across all fractions (bottom panel). Data presented are representative of two independent experiments.

To investigate how phosphorylation at T250 influences SHFL inhibition of −1PRF, we analyzed the distribution patterns of HIV-1 *gag-pol* mRNA in the polysome profiling assay. HOS-CD4/CCR5 cells expressing Myc-tagged GFP (control) or SHFL variants in a doxycycline-inducible manner were infected with VSV-G pseudotyped HIV-1 vector NL4-3 GFP and treated with doxycycline to induce protein expression. The cell lysates were subjected to polysome profiling analysis (see Materials and Methods for detailed procedure). In the GFP-expressing control cells, a peak of the *gag-pol* RNA level was detected in the light polysome fractions (Fig. 5A), which is consistent with the results published previously (34). The expression of SHFL or SHFL-T250E led to a reduction of the *gag-pol* RNA level in the light polysome fractions (Fig. 5A). In the SHFL-T250A-expressing cells, the RNA distribution pattern was very similar to that in the control cells (Fig. 5A). We also analyzed the distribution patterns of the viral *nef* RNA and host β-actin mRNA, which do not have −1PRF elements, and no obvious difference was observed (Fig. 5A).

The above results suggested that the phosphorylation at T250 may affect SHFL interaction with the translation machinery. To further investigate this possibility, we examined the association with polysomes of the SHFL variants. The 293T cells transiently expressing the SHFL variants, including SHFL, SHFL-T250A, and SHFL-T250E, were subjected to polysome profiling analysis, and the SHFL protein levels in each fraction were measured by western blotting. The wild-type SHFL was easily detected in most polysome fractions (Fig. 5B). In comparison, SHFL-T250A exhibited markedly reduced association with the polysomes, whereas the association of SHFL-T250E with the polysomes was similar to that of the wild-type protein (Fig. 5B). Collectively, these results indicate that the phosphorylation of SHFL at T250 is important for its association with polysomes.

### The T250A mutation impairs SHFL interaction with IGF2BPs

**Fig 6:**
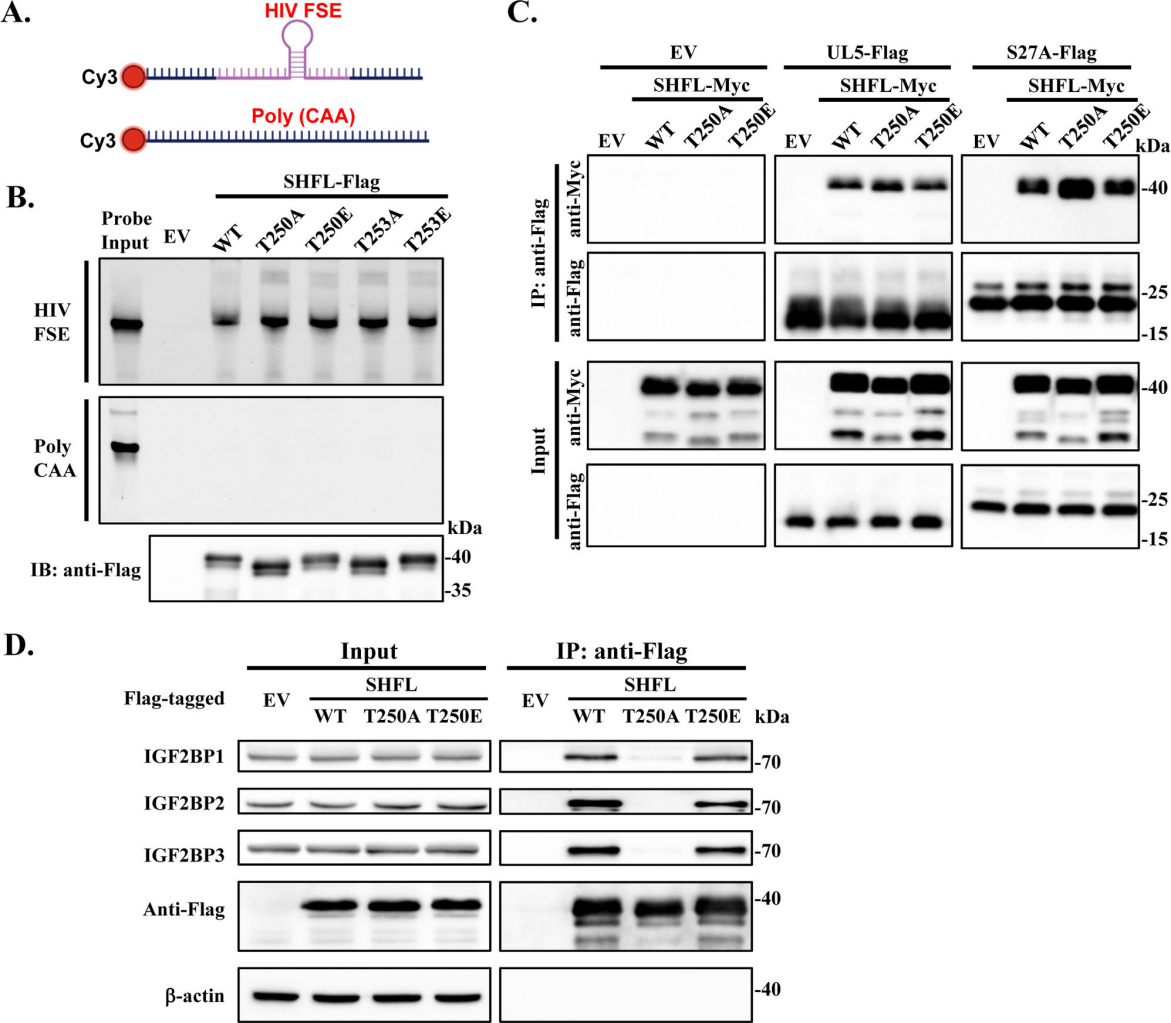
The T250A mutation does not affect SHFL interaction with target RNA or the ribosomal proteins uL5 and eS31 but impairs SHFL interaction with IGF2BPs. (**A**) Schematic representation of the RNA probes used in the *in vitro* RNA-binding assay. FSE, frameshifting element. (**B**) 293T cells were transfected with an empty vector (EV) or a plasmid expressing the Flag-tagged SHFL protein indicated. At 48 h post-transfection, SHFL proteins were immunoprecipitated using anti-Flag affinity gel and incubated with the Cy3-labeled RNA probes. The SHFL-associated RNA was detected by urea-PAGE and fluorescence detection. Data presented are representative of three independent experiments. (**C**) 293T cells were co-transfected with a plasmid expressing the Myc-tagged SHFL indicated and a plasmid expressing Flag-tagged uL5 or eS31. At 24 h post-transfection, the cell lysates were immunoprecipitated with anti-Flag affinity gel and analyzed by western blotting. Data presented are representative of three independent experiments. (**D**) Flag-tagged SHFL indicated was transiently expressed in 293T cells, immunoprecipitated with anti-Flag affinity gel and subjected to western analysis. The endogenous IGF2BP1-3 was detected using specific antibodies. Data presented are representative of two independent experiments.

To explore the mechanism by which the phosphorylation at T250 affects SHFL association with polysomes, we first assessed whether the T250A mutation influences the RNA-binding capacity of SHFL. Two Cy3-labeled RNA probes were used, HIV-1 FSE and Poly (CAA). The HIV-1 FSE probe consisted of 100-nt encompassing the frameshift element (including the slippery site and downstream stem-loop) of HIV-1. The Poly (CAA) probe was used as a negative control (Fig. 6A). Flag-tagged SHFL proteins transiently expressed in 293T cells were immunoprecipitated and then incubated with the RNA probes, followed by Urea-PAGE. The interaction of the RNA probes with the SHFL proteins was detected by the fluorescence signals precipitated with the SHFL proteins. Although none of the proteins interacted with the Poly (CAA) control RNA, they displayed comparable binding to the HIV-1 FSE probe (Fig. 6B). These results indicated that the phosphorylation of SHFL at T250 does not compromise its RNA-binding ability.

We next examined whether T250 phosphorylation affects SHFL interaction with ribosomal proteins. The ribosomal proteins uL5 and eS31 have been reported to interact with SHFL (1). Myc-tagged SHFL variants and Flag-tagged uL5 or eS31 were co-expressed in 293T cells. The interactions of the proteins were assessed by the Co-IP assay. No significant difference was observed in the interaction with uL5 or eS31 of SHFL, SHFL-T250A, and SHFL-T250E (Fig. 6C).

IGF2BPs were identified as SHFL-interacting proteins in the TAP-MS analysis (Fig. 1C). Previous studies have shown that IGF2BPs associate with polysomes (35, 36). To assess whether SHFL phosphorylation affects its interaction with IGF2BP proteins, Flag-tagged SHFL, SHFL-T250A, and SHFL-T250E were expressed in 293T cells. Co-IP analysis revealed that the T250A mutant exhibited markedly reduced interaction with the endogenous IGF2BP proteins compared with wild-type SHFL (Fig. 6D). In comparison, SHFL-T250E interacted with the endogenous IGF2BPs (Fig. 6D). These results indicate that the phosphorylation of SHFL at T250 is essential for its interaction with IGF2BP proteins and suggest the association of SHFL with the translating ribosomes is at least partially mediated by the IGF2BP proteins.

## DISCUSSION

Here, we identified CK1δ and CK1ε as kinases that interact with and phosphorylate SHFL. CK1δ/CK1ε phosphorylation of SHFL at T250 and T253 is essential for SHFL to inhibit −1PRF and to suppress HIV-1 production.

**Fig 7:**
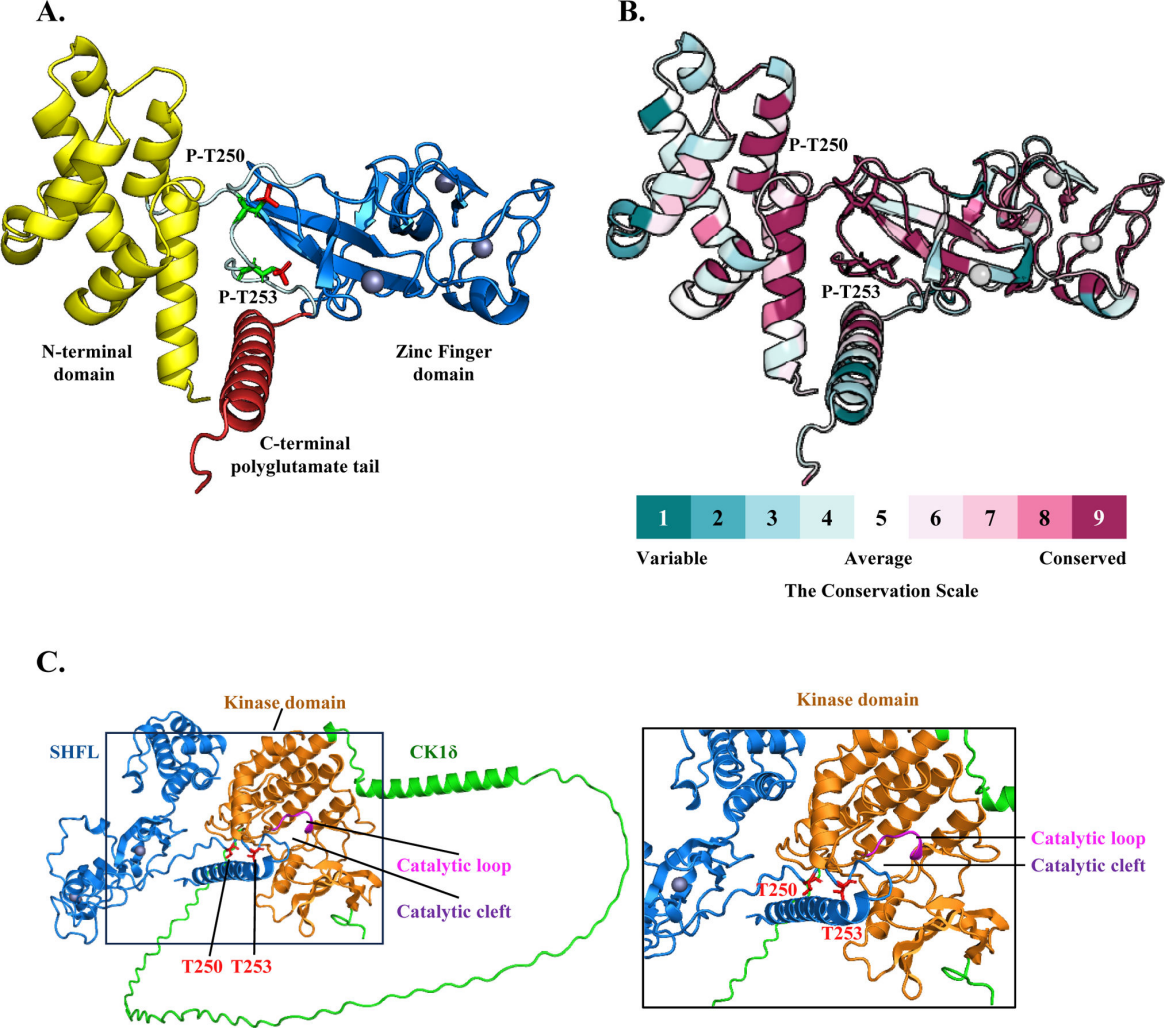
Computational analyses of SHFL structure, conservation, and its predicted interaction with CK1δ. (**A**) Predicted tertiary structure of SHFL generated by AlphaFold3, showing three distinct domains: the N-terminal domain (yellow), a zinc finger domain (blue), and the C-terminal polyglutamate tail (red). The threonine residues at T250 and T253 are represented as green sticks, the attached phosphate groups as red sticks, and zinc ions as metallic-colored spheres. Images were generated using PyMOL based on AlphaFold3-predicted coordinates. (**B**) Evolutionary conservation of SHFL residues mapped onto the AlphaFold3-predicted structure using ConSurf. Conservation scores (1–9; 1, variable; 9, conserved) calculated by Rate4Site were visualized with a cyan-to-magenta color scale. (**C**) Predicted interaction between SHFL and CK1δ revealed by AlphaFold3 multimer modeling. The left panel shows the predicted structural model illustrating the interaction between SHFL and CK1δ. SHFL is shown in blue, with T250 and T253 highlighted as red sticks. Zinc ions are represented as gray spheres. CK1δ is displayed with its overall structure in green and kinase domain in orange. The catalytic cleft and catalytic loop within the kinase domain are highlighted in magenta and pink, respectively. The right panel shows an enlarged view of the boxed region, where T250 and T253 of SHFL are positioned adjacent to the catalytic loop within the catalytic cleft of CK1δ.

How the phosphorylation of SHFL regulates its antiviral activity is not clear yet. Phosphorylation can introduce steric bulk and negative charges, alter the local physicochemical environment, and influence protein stability, conformational dynamics, and molecular interactions (37–40). Based on AlphaFold3 predictions, SHFL comprises three structured regions: an N-terminal domain, a Zinc Finger domain containing three zinc finger motifs, and a C-terminal polyglutamate (poly-E) tail. T250 and T253 are located within a predicted intrinsically disordered region embedded between these domains (Fig. 7A). Phosphorylation within such core regions could influence protein folding and structural stability (39). Consistently, ConSurf analysis, a computational method that estimates and visualizes evolutionary conservation in macromolecules (41), revealed that T250, T253, and their neighboring residues are highly conserved (Fig. 7B), suggesting that this region plays a functionally important role maintained under evolutionary constraint.

Based on our experimental data, CK1δ and CK1ε are supported as kinases that interact with and phosphorylate SHFL, consistent with their identified roles in regulating its antiviral activity. Structural modeling was performed to gain further insight into these processes. Considering the high structural similarity between CK1δ and CK1ε (27), we performed AlphaFold3 multimer prediction to evaluate the potenCOMtial interaction between SHFL and CK1δ. The predicted complex suggests a direct interaction between SHFL and CK1δ (Fig. 7C, left panel). In this model, the kinase domain of CK1δ (orange) is positioned close to SHFL (blue), and the T250 and T253 residues of SHFL (red) are located near the catalytic cleft (magenta) containing the catalytic loop (pink) of CK1δ (Fig. 7C, right panel). Given that the kinase domains of CK1δ and CK1ε are highly conserved (42), this model may also explain the observed interaction of SHFL with both kinases. Together with our biochemical data, this structural model provides computational support for a direct kinase–substrate relationship between CK1δ/ε and SHFL.

SHFL-T250A showed reduced association with polysome fractions (Fig. 5). The T250A mutation did not affect SHFL interaction with target RNA (Fig. 6B) nor its interaction with the ribosomal proteins uL5 and eS31 (Fig. 6C). However, the T250A mutation significantly reduced SHFL interaction with IGF2BP proteins, whereas the T250E mutation had little effect. These results suggest that the phosphorylation at T250 is essential for SHFL interaction with IGF2BPs. The reduced interaction between the T250A mutant and IGF2BPs, together with the known roles of IGF2BPs in mRNA localization and translation regulation (35), suggests that phosphorylation-dependent association with IGF2BPs is important for SHFL to achieve spatial and functional coupling with the translation machinery, as similar potential recruitment mechanisms have been suggested (43, 44). Further investigation is needed to decipher the precise mechanism underlying this process.

In HIV-1-infected control cells, polysome profiling revealed a peak signal of *gag-pol* mRNA in the light polysome fractions, consistent with previous observations (34). The lower ribosome density in these fractions may permit RNA structures to refold or remain intact, thereby promoting ribosome pausing and enabling −1PRF. Upon SHFL expression, the *gag-pol* mRNA peak in the light polysome fractions was selectively diminished, suggesting that SHFL effectively suppressed −1PRF within this region. SHFL has been reported to promote ribosome dissociation by recruiting eukaryotic release factors at the frameshifting site (1). The reduction of *gag-pol* RNA levels in the polysome fractions is in line with the notion that SHFL caused premature ribosome dissociation. SHFL-T250E exhibited a polysome distribution pattern of *gag-pol* mRNA similar to the wildtype SHFL, whereas SHFL-T250A mirrored the GFP control. These mRNA distribution patterns are consistent with the polysome association pattern of SHFL; the wild-type SHFL and the T250E mutant exhibited comparable association with the polysome fractions, whereas the T250A mutant showed a markedly reduced distribution in the polysome fractions. These results emphasize the critical role of T250 phosphorylation in enabling SHFL association with polysomes, which is required for its ability to suppress −1PRF.

In the polysome profiling analysis, SHFL was enriched in ribosomal fractions, including the actively translating polysomal fractions (1–5) and the non-translating ribosomal fractions (80S/60S/40S; 5-8), with a smaller portion detected in the free fractions (9, 10) (Fig. 5B). This distribution pattern is consistent with SHFL interaction with ribosomes. SHFL detected in the free fractions may represent the protein partially phosphorylated or unphosphorylated at T250 and T253, as CK1δ/ε-mediated phosphorylation is often incomplete due to limited kinase accessibility and the reversible nature of this modification (45–47). The T250A mutation impaired SHFL interaction with IGF2BPs, resulting in reduced association with polysomes and increased accumulation in the non-translating ribosomal fractions. The phosphomimetic T250E mutant behaved more like the WT protein in the polysome fractions, which is in line with its antiviral activity. More T250E was detected in fractions 5–7 than the WT protein. One possible explanation is that more T250E was detected in fractions 5–7 because less T250E was detected in fractions 9 and 10, which are supposed to contain only the free SHFL protein. T250E could be considered fully phosphorylated, while the WT protein phosphorylation is expected to be heterogeneous, and thus there is always a fraction of the WT protein being unphosphorylated and thereby not associated with the ribosome.

In summary, our results indicate that CK1δ/ε phosphorylation at T250 is essential for SHFL to engage the translational machinery and to suppress −1PRF, thereby restricting viral replication. These findings highlight phosphorylation at T250 as a molecular requirement for the antiviral activity of SHFL.

## MATERIALS AND METHODS

### Plasmids and cell culture

The coding sequences of CK1δ (NM_001893.6), CK1δ2 (NM_139062.4), and CK1ε (NM_152221.3) were PCR-amplified from 293T cell-derived cDNA and cloned into the pCMV-HA-Flag (pCMV-HF) expression vector as described previously (48). Site-directed mutagenesis of pCMV-HF-SHFL or pLPCX-SHFL-Myc was performed as described previously (1). The dual-luciferase reporters pDual-HIV(−1) and pDual-HIV(0) were constructed by inserting the HIV-1 -1PRF signal sequence or the modified control sequence between the Renilla luciferase (Rluc) and Firefly luciferase (Fluc) coding regions, as described by Léa Brakier-Gingras (49).

To generate doxycycline-inducible SHFL-expressing cell lines for tandem affinity purification (TAP) assays, the coding sequence of HA-Flag-SHFL was cloned into the lentiviral vector pTRIPZ-puro (Horizon). The resulting vector was transduced into HEK293 cells. To express GFP-Myc, SHFL-Myc, and its mutants in HOS-CD4/CCR5 cells in a doxycycline-inducible manner, the coding sequences were cloned into a modified pTRIPZ-mCherry vector, in which the puromycin resistance gene was replaced with the mCherry fluorescence marker (50). CK1δ/ε knockout cell lines were generated using sgRNA sequences cloned into the LentiCRISPRv2-puro vector, which was kindly provided by Dr. Mingzhao Zhu of the Institute of Biophysics, Chinese Academy of Sciences.

HEK293T, HEK293, and HOS-CD4/CCR5 were cultured in Dulbecco’s modified Eagle medium (DMEM, Invitrogen) supplemented with 10% fetal bovine serum (Gibco). Puromycin-resistant cell lines were selected in medium containing 5 µg/mL puromycin (Sigma-Aldrich) and maintained in medium containing 1 µg/mL puromycin.

Plasmid DNA transfection was carried out using Xpregen following the manufacturer′s instructions (Beijing Yu-Feng Biotechnology, Cat no. ND01). Small interfering RNA (siRNA) transfection was performed with Lipofectamine 2000 (Thermo Fisher Scientific) following the manufacturer’s recommended protocol.

### Antibodies

To generate antisera specifically recognizing SHFL phosphorylated at T250 and T253, a synthetic phosphopeptide (SGS-pT-VA-pT-SLS-C) conjugated to Keyhole Limpet Hemocyanin (SciLight Biotechnology) was used to immunize rabbits. The antisera were harvested following standard immunization protocols and validated for specificity before use. SHFL-specific polyclonal antibodies were kindly provided by Dr. Xuan Yifang. The p24-specific antibody was kindly provided by Prof. Yongtang Zheng (51). Commercial antibodies used in this study included: mouse monoclonal anti-β-actin (GSGB-BIO, China; TA-09), mouse monoclonal anti-Myc (9E10, Santa Cruz Biotechnology; sc-40), mouse monoclonal anti-Flag M2 (Sigma-Aldrich; F3165), rabbit polyclonal anti-IGF2BP1 (Proteintech; 22803-1-AP), rabbit monoclonal anti-IGF2BP2 (Abclonal; A5189), rabbit monoclonal anti-IGF2BP3 (Abclonal; A23295), anti-CK1ε (Abcam; ab302638), and rabbit polyclonal anti-CK1δ (Proteintech; 14388-1-AP).

### TAP-MS and protein immunoprecipitation assays

For TAP-MS analysis, HEK293 cells expressing HA-Flag-SHFL in a doxycycline-inducible manner were treated with 1 µg/mL doxycycline for 24 h to induce protein expression, washed twice with ice-cold PBS, and lysed in Co-IP buffer (150 mM NaCl, 25 mM Tris-HCl pH 7.4, 2 mM MgCl₂, 0.5% IGEPAL CA-630 [Sigma-Aldrich]) supplemented with Complete EDTA-free protease inhibitor cocktail (Sigma-Aldrich), PhosSTOP phosphatase inhibitor (Sigma-Aldrich) and 250 U/mL Benzonase (Sigma-Aldrich). The cell lysates were clarified through centrifugation at 4°C for 15 min. For Flag-tag affinity purification, anti-Flag M2 affinity gel (Sigma-Aldrich) was washed three times with PBS, incubated with the clarified cell lysates at 4°C for 2 h with gentle rotation, followed by washing three times with PBS. Proteins were eluted by incubation with Flag elution buffer (200 µg/mL 3 × FLAG peptide [SciLight] in Co-IP buffer) at 4°C for 30 min. After centrifugation at 5,000 × *g* for 1 min, the eluates were transferred to new tubes. For HA-tag affinity purification, anti-HA magnetic beads (Thermo Fisher Scientific) were washed three times with PBS, incubated with the above eluates at 4°C for 2 h with gentle rotation, and collected using a magnetic stand. Proteins were eluted with acid elution buffer (0.1 M Glycine-HCl, pH 2.5) at room temperature for 10 min. Eluates were neutralized immediately using neutralization buffer (15 µL per 100 µL eluate; 1 M Tris-HCl, pH 8.5, 1.5 M NaCl), aliquoted, snap-frozen in liquid nitrogen, and stored at −80°C for subsequent western blotting and mass spectrometry analyses. For mass spectrometry analysis, protein bands visualized by silver staining were excised and analyzed by the National Center for Protein Sciences as described previously (1). The TAP-MS data have been deposited in the iProx database (ProteomeXchange accession number PXD068760) (52).

Co-immunoprecipitation (Co-IP) assays using Flag-tagged SHFL variants were performed as described in the TAP-MS analysis. Briefly, 293T cells were transfected with plasmids expressing Flag-tagged SHFL variants. At 24 h post-transfection, the cells were lysed in Co-IP buffer, and lysates were subjected to immunoprecipitation using anti-Flag M2 affinity gel. The immunoprecipitants were analyzed by Western blotting using the indicated antibodies to detect interacting proteins.

For endogenous SHFL immunoprecipitation and phosphorylation analysis, THP-1 cells were treated with interferon-alpha for 24 h, in the presence or absence of the CK1δ/ε inhibitor PF-670462 at 40 µM. Cells were lysed in RIPA buffer (20 mM Tris-HCl [pH 7.4], 150 mM NaCl, 1% IGEPAL CA-630 [Sigma-Aldrich], 1% sodium deoxycholate), supplemented with Complete EDTA-free protease inhibitor cocktail (Sigma-Aldrich) and PhosSTOP phosphatase inhibitor (Sigma-Aldrich). Lysates were clarified by centrifugation and incubated with SHFL-specific polyclonal antibodies and protein G beads (Amersham Pharmacia) at 4°C for 2 h. The immunoprecipitants were analyzed by western blotting using antisera specific for SHFL phosphorylated at T250 and T253.

### Downregulation of CK1

To generate CK1δ or CK1ε knockout cells, HEK293 cells were transduced with VSV-G–pseudotyped lentiviral vectors based on LentiCRISPRv2-puro expressing sgRNAs targeting CK1δ or CK1ε at CDS region, two sgRNAs were designed for each gene. Given that CK1δ has two functional isoforms (27), the sgRNAs targeting CK1δ were selected to disrupt both CK1δ and CK1δ2 by recognizing sequences at the N terminus. A non-targeting sgRNA (sgNC) was included as a control. Transduced cells were selected in medium containing 5  µg/mL puromycin, and the pool of resistant cells was used. Knockout efficiency was confirmed by western blotting using CK1δ- or CK1ε-specific antibodies. The sgRNA sequences used are listed below.

sgNC: 5′-AAATGTGAGATCAGAGTAAT-3′

sgCSNK1D-1: 5′-GGACTACAACGTCATGGTGA-3′

sgCSNK1D-2: 5′-TGAGAGTCGGGAACAGGTAC-3′

sgCSNK1E-1: 5′-CCGCAAATTCAGCCTCAAGA-3′

sgCSNK1E-2: 5′-GTCCTTCGGAGATATCTACC-3′

To knock down CK1ε in CK1δ knockout cells, the cells were transfected twice with scrambled siRNA (SCR) or CK1ε-specific siRNA (GenePharma), with or without a CK1ε rescue construct, using Lipofectamine 2000 (Thermo Fisher Scientific) according to the manufacturer’s instructions. In the second-round transfection, plasmids required for subsequent assays were co-transfected. The siRNA sequences have been previously described (4) and are listed below.

Scrambled siRNA (SCR): 5′- UUCUCCGAACGUGUCACGUTT-3′

siCSNK1E: 5′-CCUCCGAAUUCUCAACAUATT-3′

To confirm the specificity of CK1δ and CK1ε downregulation, rescue expression constructs of CK1δ and CK1ε that were not targeted by the CK1δ sgRNAs or CK1ε siRNA were generated. The nucleotide sequences recognized by the CK1δ sgRNAs and CK1ε siRNA were synonymously mutated and cloned into expression vectors. The mutated sequences of CK1δ were 5′-GGCGATTATAATGTGATGGTCATGGAA-3′ and 5′-CTGCGGGTGGGCAATAGATATAGA-3′, and the mutated sequence of CK1ε was 5′-CCTAGCGAGTTTAGCACCTAT-3′.

### Polysome profiling

To analyze the association of SHFL with ribosomes, 293T cells were transfected with an empty vector or a plasmid expressing Myc-tagged SHFL. At 24 h post-transfection, the cells were treated with 100 µg/mL cycloheximide (INALCO), lysed in polysome lysis buffer (150 mM KAc, 5 mM MgCl₂, 50 mM HEPES pH 7.4, 0.5% IGEPAL CA-630, 100 µg/mL cycloheximide). The cell lysates were clarified through centrifugation for 15 min at 4°C and layered onto a 10%–50% sucrose gradient and centrifuged at 36,000 rpm for 3.3 h at 4°C (Hitachi P40ST rotor). Polysome profiles were monitored by absorbance at 260 nm, and fractions were collected. To detect the protein levels, 100  µL of each fraction was mixed with 20  µL of SDS-PAGE loading buffer and analyzed by western blotting.

To analyze the RNA distribution patterns in the polysome profiling assay, HOS-CD4/CCR5 cells expressing Myc-tagged GFP or SHFL in a doxycycline-inducible manner were infected with VSV-G pseudotyped NL4-3 GFP for 3 h. Cells were then treated with doxycycline at 1  µg/mL to induce protein expression. At 24 h postinfection, cells were lysed with polysome lysis buffer and subjected to polysome profiling analysis as described above. From each fraction, 250  µL was collected and mixed with 750  µL TRIzol-LS reagent (Invitrogen). The *in vitro* transcribed Fluc RNA was added to each sample before RNA extraction to serve as a control for sample handling. Reverse transcription was performed using random primers (Takara), followed by SYBR Green-based qPCR (SuperReal PreMix Plus, Tiangen) with primers specific for HIV-1 Gag-Pol, Nef, and Fluc mRNA. Quantification of Gag-Pol, Nef, and Fluc mRNA was performed using the standard curve method. The relative level of Gag-Pol, Nef, or ACTB mRNA was normalized with the Fluc RNA level. The qPCR primer sequences are listed below.

qGag-Pol-F: 5′-AAAGGCACAGCAAGCAGCAG-3′

qGag-Pol-R: 5′-ACCATTTGCCCCTGGAGGTT-3′

qNef-F: 5′-ACAGTCAGACTCATCAAGCTTCTCT-3′

qNef-R: 5′-CGGGTCCCCTCGGGATTG-3′

qFLuc-F: 5′-CCAGGGATTTCAGTCGATGT-3′

qFLuc-R: 5′-AATCTGACGCAGGCAGTTCT-3′

qACTB-F: 5′-CACCATTGGCAATGAGCGGTTC-3′

qACTB-R: 5′-AGGTCTTTGCGGATGTCCACGT-3′

### *In vitro* RNA binding assay

293T cells were transiently transfected with constructs expressing Flag-tagged SHFL proteins. At 48 h post-transfection, cell lysates were immunoprecipitated with anti-Flag affinity gel. The resins were washed three times with PBS and then incubated with Cy3-labeled RNA probe (GenScript) in RNA-binding buffer (25  mM Tris-HCl pH 7.5, 150  mM NaCl, 1  mM EDTA, 10  µM ZnCl₂, 0.1  mg/mL yeast tRNA [Invitrogen], and SUPERase·In [Thermo Fisher Scientific]) with intermittent agitation at 26  °C for 30  min. The resins were washed three times with PBS and suspended in 50 µL PBS. An aliquot (10 µL) was analyzed by TBE-urea gel electrophoresis. The Cy3 fluorescence was detected using a Typhoon imaging system. Another aliquot (10–20 µL) of the sample was analyzed by western blotting to ensure that comparable amounts of the SHFL proteins were used. The sequences of the RNA probes have been described previously (1).

### Virus production assays

To evaluate SHFL inhibition of HIV-1 production, 293T cells were co-transfected with the SHFL-expressing construct pLPCX-SHFL-Myc or the control construct expressing GFP-Myc, together with pNL4-3 Env-Luc. At 48 h post-transfection, the cells were lysed. The expressions of the viral proteins and SHFL were analyzed by western blotting, and the Firefly luciferase activity was measured using a luciferase assay system (Promega). The culture supernatants were used to infect recipient HOS-CD4/CCR5 cells. At 48 h postinfection, luciferase activity in the recipient cells was measured and normalized with the luciferase activity in the producer cells. Fold inhibition of SHFL against HIV-1 production was calculated as the normalized luciferase activity from the control cells divided by that from the SHFL-expressing cells.

### Statistical analysis

Statistical analyses were performed using GraphPad Prism and Proteome Discoverer. Unless otherwise specified, data are presented as arithmetic mean ± standard deviation (SD) from three independent experiments. For pairwise comparisons, *P*-values were determined using a two-tailed paired Student’s *t*-test. For multiple group comparisons, one-way analysis of variance (ANOVA), followed by Tukey’s post hoc test, was applied. The number of replicates and additional details relevant to data reliability are provided in the corresponding figure legends.

## Data Availability

TAP–MS proteomics raw data files and label-free quantification file are deposited with the ProteomeXchange Consortium via iProX under accession PXD068760 (https://proteomecentral.proteomexchange.org/cgi/GetDataset?ID=PXD068760).
